# Supplementary material for: Content-rich biological network constructed by mining PubMed abstracts
Source: BMC Bioinformatics. 2004 Oct 8;5:147. doi: 10.1186/1471-2105-5-147 (PMC528731; doi:10.1186/1471-2105-5-147)
Supplement: Additional File 2 — The original results of the above study (non-essential files are deleted to keep the file size under the limit set by BMC bioinformatics). [file 1471-2105-5-147-S2.bz2 › chilibotAdditionalFile2/dip05/183ID7957082E623/html/IL3_CSF2RB.html]

 


 **IL3** and **CSF2RB** 
  
Found 1 abstracts in PubMed, retrieved 1.  
 

 What does Google say? 
 PDF only 
| .edu only 

---

- J Biol Chem, 1992   **Chromosomal localization and organization of the murine genes encoding the beta subunits AIC2A and AIC2B of the interleukin 3 **[IL3]**, granulocyte macrophage colony stimulating factor, and interleukin 5 receptors.** .
  Chromosomal genes for two mouse homologous beta subunits AIC2A and AIC2B of the interleukin 3 **[IL3]**, granulocyte macrophage colony stimulating factor, and interleukin 5 receptors were characterized.
  Both AIC2A and AIC2B genes were present on a 250 kilobase MluI restriction fragment and were mapped on murine chromosome 15 these loci were provisionally designated as Il3rb **[CSF2RB]** 1 AIC2A and Il3rb **[CSF2RB]** 2 AIC2B , closely linked to the c sis locus.
  Both genes consist of 14 exons and span about 28 kb each.
  The major transcription initiation sites of both genes were mapped at 194 bp from the initiation codon.
  These genes are 95% identical up to 700 bp from the transcription initiation sites.
  Potential recognition sequences for hemopoietic transcription factors including GATA 1 and PU.1 in addition to a TATA like sequence are present in the 5 flanking region.
  A stretch of 20 bp including the initiation site is homologous to the corresponding region of the erythropoietin receptor and the interleukin 7 receptor genes and to the initiator sequence of the adeno associated virus P5 promoter, suggesting a possible role in transcription initiation.
  Comparison of the exon intron boundaries of AIC2A and AIC2B genes with those of other members of the cytokine receptor superfamily reveals a conserved evolutionary structure.
  Isolation of various forms of AIC2 cDNAs reveals differential splicing of the transcripts.
